# Fruit physical characteristics, proximate, mineral and starch characterization of FHIA 19 and FHIA 20 plantain and FHIA 03 cooking banana hybrids

**DOI:** 10.1186/s40064-016-2465-1

**Published:** 2016-06-21

**Authors:** George Amponsah Annor, Prudence Asamoah-Bonti, Esther Sakyi-Dawson

**Affiliations:** Department of Nutrition and Food Science, University of Ghana, P O Box LG 134, Legon-Accra, Ghana

**Keywords:** Plantain, Cooking banana, Proximate, Starch

## Abstract

Cooking banana and plantain (*Musa* spp. AAB and ABB groups), have over the years been affected by pest and diseases, resulting in various organizations developing disease resistant hybrids with superior agronomic potential. The characteristics of these improved varieties needs to be studied to ascertain their suitability for use in various food systems. This study aimed at evaluating the physical characteristics, proximate and minerals composition, and characterizing the starch of plantain and a cooking banana hybrid release by *Fundación Hondureña de Investigación Agrícola* (FHIA), and comparing them to a local landrace in Ghana. FHIA 19 and FHIA 20 plantain, *Apentu pa* (a local landrace) and FHIA 03 cooking banana hybrid were used for the study. Their physical characteristics, proximate and mineral composition were determined at the proximal, midsection and distal hand positions. Starch granules and cells were then examined under light microscope. Ranges obtained for protein content for FHIA 20, FHIA 03 and FHIA 19 were 3.01–3.40, 2.66–2.91 and 2.81–2.91 %. Potassium was found to be the most abundant mineral in all the cultivars. The highest mean value of 982.5–1013.76 mg/100 g was obtained for FHIA 19. There were significant differences (p < 0.05) in the proximate and mineral composition of the varieties, however no significant difference exited between the hand positions. The largest starch granule size was found in FHIA 19 hybrid. FHIA 03 was also composed predominantly of two types: longitudinal and rounded granules with each type grouped together. The new plantain hybrids compared very well with the local landrace hence making them suitable to be incorporated into local food systems.

## Background

Plantain and cooking banana (*Musa* spp. AAB and ABB groups) is cultivated mainly as a carbohydrate staple in many developing countries, especially in Africa (IITA 2012). According to the Food and Agriculture Organization of the United Nations statistical division (FAOSTAT 2013), 106,714,205 tonnes and 37,877,805 tonnes of banana and plantains was produced worldwide, with about 16 % banana and 72 % plantain respectively produced in Africa. Banana, cooking banana and plantain exports are essential for the economies of Central and South America and the West Indies (Sakyi-Dawson et al. 2008). Production levels of plantains and cooking banana are however affected by several factors. Notable amongst these factors are diseases such as the black sigatoka (*Mycosphaerella fijiensis*), a serious leafspot disease (Stover and Simmonds 1978). To reduce this significant effects of diseases on the production levels of plantain and cooking bananas, institutions such as the International Institute of Tropical Agriculture (IITA) Nigeria and Fundación Hundureña de Investigación Agricola (FHIA), Honduras have developed several cultivars of plantain and banana which are disease and pest resistant, high yielding and with good postharvest qualities that are being tested and or distributed to farmers in plantain growing areas. The need to characterize these new and improved varieties to assess their suitability in various food systems, and their eventual adoption in various diets is very important. This study aimed at evaluating the fruit quality characteristics, proximate and mineral composition and starch characteristics of new and promising plantain and cooking banana introduced into Ghana by FHIA in Honduras and compared to *Apantu pa* plantain; a local cultivar.

## Results and discussions

### Physical characteristics

The physical characteristics of the plantain and cooking banana cultivars are summarized in Tables 1 and 2. The bunch weights were notably heavier than that observed for tetraploid plantain hybrids TMPx 1658-4 and TMPx 548-9 and triploid cooking banana landrace Fougamou respectively (Ferris et al. 1996). The cooking banana hybrid FHIA 03 was the heaviest, though the shortest amongst the three varieties studied. A weight of 25.3 kg was recorded for the FHIA 03 compared to 22.7 and 22.6 kg of the FHIA 19 and FHIA 20 respectively. Compared to the *Agbagba* plantain landrace which has an average of 15 fingers per bunch (Ferris et al. 1996), the FHIA 19 and FHIA 20 plantain hybrids had fewer fingers per bunch. Fruits of the cooking banana hybrid FHIA 03 were shorter and bigger compared to the plantains hybrids. In many West African markets, the shorter finger length FHIA 03 cooking banana would be associated with sweet dessert bananas. This is likely to reduce its market value as a cooking cultivar. The number of hands on a bunch was similar for the three varieties. The pulp of the FHIA 19 plantain hybrid was firmer than that of the FHIA 20 which was in turn firmer than the pulp of the FHIA cooking banana hybrid (Table 3). The pulp of bananas having higher contents of pectin has been shown to be softer than plantains (Dadzie 1993). The firmness of the plantain hybrids is obviously an advantage in post harvest management. Loss of firmness during ripening leads to higher incidence of mechanical damage, making the ripened hybrids more difficult to manage. The results also indicated that the samples from the distal hand positions of the two plantain and cooking banana hybrids has firmer pulps as compared to samples from the proximal and midsection hand positions.

**Table 1:** Physical characteristics of plantain and cooking banana cultivars

Characteristics	Cultivars	Mean	Minimum	Maximum
Bunch weight (kg)	FHIA03	25.3 ± 2.0	22.0	28.0
	FHIA20	22.6 ± 1.4	20.0	25.0
	FHIA19	22.7 ± 2.4	20.0	26.0
	Apantu pa	7.8 ± 2.6	7.2	9.8
No of hands on a bunch	FHIA03	9.0 ± 0.2	8.0	10.0
	FHIA20	8.9 ± 0.9	8.0	10.0
	FHIA19	8.9 ± 0.9	8.0	10.0
	Apantu pa	5.0 ± 0.8	4.0	6.0

Mean values (g/100 g, dry matter basis) from triplicate analysis ± standard deviation

**Table 2:** Fruit characteristics of plantain and cooking banana cultivars

Characteristics	Hand position	Cultivars			
		FHIA 03	FHIA 20	FHIA 19	Apantu pa
Fruit width (cm)	Proximal	16.0 ± 0.2	13.2 ± 0.6	14.0 ± 0.4	16.0 ± 0.4
	Midsection	15.3 ± 0.0	13.1 ± 0.0	13.0 ± 0.0	15.3 ± 0.0
	Distal	13.3 ± 0.2	11.3 ± 0.2	11.5 ± 0.1	14.4 ± 0.3
Fruit length (cm)	Proximal	17.5 ± 0.1	22.5 ± 0.2	23.0 ± 0.2	26.5 ± 0.4
	Midsection	16.5 ± 0.2	21.0 ± 0.6	21.5 ± 0.1	25.0 ± 0.2
	Distal	15.5 ± 0.1	17.5 ± 0.1	17.5 ± 0.0	17.5 ± 0.0

Mean values (g/100 g, dry matter basis) from triplicate analysis ± standard deviation

**Table 3:** Pulp firmness, pulp colour and starch content of plantain and cooking banana hybrids

Cultivar	Hand position	Pulp to peel ratio	Pulp firmness (g/s)	Starch content (%)
FHIA 03	Proximal	0.93	2171.0	74.9 ± 0.0
	Midsection	0.91	2156.7	74.7 ± 0.0
	Distal	0.81	2152.6	74.4 ± 0.1
FHIA 20	Proximal	1.14	4163.1	79.9 ± 0.2
	Midsection	1.10	4158.6	79.3 ± 0.0
	Distal	0.90	4148.6	79.1 ± 0.0
FHIA 19	Proximal	1.27	4756.7	81.5 ± 0.0
	Midsection	1.24	4730.7	81.6 ± 0.0
	Distal	1.20	4751.4	80.3 ± 0.1
Apantu pa	Proximal	1.68	5742.9	85.0 ± 0.1
	Midsection	1.45	5720.6	84.3 ± 0.2
	Distal	1.21	5715.1	85.0 ± 0.0

Mean values (g/100 g, dry matter basis) from triplicate analysis ± standard deviation

The colour of plantains and cooking bananas probably contributes more to the assessment of quality by the consumer than any other single factor. In some West African countries, if the pulp colour of plantain and cooking bananas is white, consumers relate that to immaturity, howerver, if the pulp is orange/yellow it indicates that the fruit is mature (Dadize 1998). The FHIA 03 cooking banana pulp was lighter in colour than the two plantain hybrids (Table 4), which were more yellow. The yellow colour may be due the carotenoids in the plantain.

**Table 4:** Pulp colour of plantain and cooking banana hybrids

Cultivar	L	a*	b*
FHIA 03	84.5 ± 0.1	1.9 ± 0.1	64.4 ± 0.1
FHIA 20	56.5 ± 0.2	15.0 ± 0.0	68.1 ± 0.2
FHIA 19	56.2 ± 0.2	14.0 ± 0.1	68.0 ± 0.1
Apantu pa	53.1 ± 0.5	16.9 ± 0.1	80.4 ± 0.3

Mean values (g/100 g, dry matter basis) from triplicate analysis ± standard deviation

The *Apantu pa* Landrace plantain had a higher percentage pulp per finger than the plantain and cooking banana hybrids, with a range of 1.7–1.2. FHIA 20 and FHIA 19 had 0.9–1.1 and 1.2–1.3 respectively with FHIA 03 cooking banana having a significantly lower (p < 0.05) pulp to peel ratio of 0.8–0.9 (Table 3). There was also a significant reduction in % pulp from the proximal to the distal sections of the bunches of all the cultivars.

### Proximate composition

The results of the proximate composition are summarized in Table 5. The moisture content was determined on the fresh plantain and cooking banana samples. It is clear from the table of results that the two plantain hybrids FHIA 19 and FHIA 20 have higher moisture contents than the cooking banana FHIA 03. Comparing the three new varieties to the *Apentu pa*, the results showed that the *Apentu pa* had the lowest moisture content. The moisture contents of the varieties studied were significantly different, however with respect to the hand positions, the differences in moisture contents were not significantly different. The moisture contents of the samples which is inversely related to its dry matter have been shown to be a useful quality-screening attribute. Sensory evaluation of both boiled and fried *musa* fruit showed that the higher the dry matter contents, the better the eating quality. Selection of new progeny based on dry matter content provides an efficient way of eliminating materials with low quality fruit (Ferris et al. 1996). It has also been reported that dry matter decreases with maturation (Trease and Evans 1989). This increase is due to carbohydrate utilization during maturation and osmotic transfer of water from the peel to the pulp. The osmotic transfer occurs due to the marked difference in osmotic pressure between peel and pulp during maturation (Loeseck 1950). The fat contents of the plantain and cooking banana samples were generally low. Crude fat contents of the FHIA 19, FHIA 20 plantain hybrids and the cooking banana hybrid FHIA 03 were 0.08, 0.12 and 0.16 % respectively. These values are lower than that reported by earlier (Giami and Alu 1993). The difference in the crude fat content of the plantain and cooking banana hybrids may be due to the differences in varieties and geographical factors (Emaga et al. 2007). Even though the fat contents were generally low, differences between the varieties were significantly different.

**Table 5:** Proximate composition of Plantain and cooking banana cultivars

Cultivar	Hand position	Moisture (%)	Protein (%)	Ash (%)	Fibre (%)	Fat (%)
FHIA 03	Proximal	74.3 ± 0.2	2.7 ± 0.1	2.8 ± 0.1	4.3 ± 0.0	0.0
	Mid section	73.5 ± 0.1	2.9 ± 0.1	2.9 ± 0.1	5.6 ± 0.0	0.3 ± 0.0
	Distal	75.0 ± 0.4	2.8 ± 0.4	3.1 ± 0.2	4.2 ± 0.0	0.2 ± 0.0
FHIA 20	Proximal	66.7 ± 0.1	3.0 ± 0.1	2.6 ± 0.1	5.4 ± 0.0	0.2 ± 0.0
	Mid section	66.7 ± 0.1	3.4 ± 0.6	2.9 ± 0.3	6.0 ± 0.1	0.0 ± 0.0
	Distal	67.0 ± 0.1	3.1 ± 0.1	2.9 ± 0.2	6.7 ± 0.2	0.2 ± 0.0
FHIA 19	Proximal	65.2 ± 0.4	2.8 ± 0.1	2.9 ± 0.2	5.5 ± 0.0	0.0
	Mid section	65.0 ± 0.6	2.9 ± 0.1	2.5 ± 0.3	6.2 ± 0.0	0.2 ± 0.1
	Distal	65.6 ± 0.1	2.8 ± 0.1	3.0 ± 0.3	5.9 ± 0.0	0.1 ± 0.0
Apantu pa	Proximal	54.3 ± 0.2	2.5 ± 0.1	2.0 ± 0.2	4.0 ± 0.0	0.2 ± 0.0
	Mid section	55.0 ± 0.2	2.8 ± 0.1	2.0 ± 0.3	4.2 ± 0.0	0.2 ± 0.0
	Distal	53.1 ± 0.5	2.9 ± 0.1	2.0 ± 0.2	3.9 ± 0.1	0.2 ± 0.0

Mean values (g/100 g, dry matter basis) from triplicate analysis ± standard deviation

The fibre concentrations of all the samples studied were below 7 %. The cooking banana hybrid FHIA 03 was found to have the lowest fibre content.

The crude protein concentrations of the plantain varieties were higher than the cooking banana. The FHIA 20 plantain hybrids recorded the highest protein content. Crude protein of plantain is lower than other starchy staples. About 5.6 g/100 g has been reported for sweet potatoes (Bradbury and Halloway 1988), 6.4–9.6 g/100 g for yams (Agbor-Egbe and Treche 1995) and about 1.7 g/100 g reported for cassava (Gomez and Valdivieso 1983). One hundred grams of the plantains and cooking banana hybrid can supply only 6 % of the RDA for protein.

More than 74 % of the plantain and cooking bananas was composed of starch. The starch content of the plantains was higher that the cooking banana (Table 3). Differences in the starch contents of various plantain and cooking banana cultivars have been reported. Plantain hybrids TMPx 1658-4 and TMPx-148-9 and TMPx 612-74 have been reported to have starch contents of 74, 72, and 70 % (dry weight) and plantain landrace *Agbagba* and *ObinoL’Ewai* have starch content of 75 and 73 % (dry weight)Cooking bananas *Pelipita Fougamou* and *Cardaba* have 73, 69, and 74 % (dry weight) respectively (Ferris et al. 1996).

The *Apantu pa* landrace plantain had the highest amylose (32.65 %) content will therefore produce a more viscose paste and is likely to retrograde faster when cooked. FHIA 19 and FHIA 20 recorded 28.1 and 26.1 % amylose respectively. FHIA 03 had the lowest quantity of amylose (25.1 %). This different amylose contents will affect the quality of certain processed forms of plantains like the traditional *fufu* which is made by pounding boiled plantains.

### Mineral composition

Considerable variations in mineral concentration in plants have been generally observed. Though little is known regarding the environmental and physiological processes that regulate the uptake of minerals in plants, the influence of species, and concentration of minerals in the soil and age of plant have been reported. Plantains and cooking bananas have been observed to accumulate potassium. The high levels of potassium and low levels of sodium obtained in this study will make these cultivars useful in low sodium diets. Potassium was found to be the most abundant mineral in all the cultivars (Table 6). The highest mean value (mg/100 g dry weight) of 982.5–1013.8 was obtained for FHIA 19. FHIA 03 had 994–1001; FHIA 20 had 726.4–817.1 whilst *Apantu pa* had the lowest value of 769.0–773.2. Mean values (mg/100 g dry weight) obtained for sodium were rather low for all the cultivars. Values obtained for FHIA 03, FHIA 20, FHIA 19, and *Apantu pa* were 2.0–2.4, 1.2–3.1, 1.6–3.1, and 2.0–2.1 respectively. The mean values of phosphorus which was also quite high indicates that 100 g of the plantain and cooking banana hybrids can supply about one quarter of the RDA for phosphorus which is 800 mg, whilst *Apantu pa* landrace plantain can supply 19 %. The appreciable amounts of calcium and magnesium but low levels of iinc and iron obtained in the samples indicate that 100 g of the plantains and cooking banana hybrids can supply about 4.5 and 1.5 % RDA of calcium respectively, while *Apantu pa* can supply 2 %. One hundred grams of the plantains and cooking banana hybrids can supply about 23 % of the RDA for magnesium, 2 % of the RDA for zinc, and 22 % of the RDA foriron. It is important to note that the iron present in plantain is completely utilized by the human body when ingested (Loeseck 1950).

**Table 6:** Mineral composition of plantain and cooking banana cultivars

Cultivar	Hand position	Na	K	Ca	Mg	Fe	Zn	P_2_O_5_
FHIA 03	Proximal	2.1 ± 0.3	994.0 ± 4.2	12.6 ± 0.4	33.1 ± 1.2	2.7 ± 0.1	0.3 ± 0.0	220.0 ± 2.4
	Mid section	2.0 ± 0.3	997.0 ± 5.3	13.4 ± 0.5	35.0 ± 2.1	3.1 ± 0.0	0.2 ± 0.0	213.0 ± 2.2
	Distal	2.1 ± 0.3	1001.0 ± 5.1	13.4 ± 0.3	34.3 ± 0.6	2.2 ± 0.1	0.3 ± 0.0	211.0 ± 3.2
FHIA 20	Proximal	3.0 ± 0.2	817.1 ± 4.3	22.6 ± 0.3	72.8 ± 3.3	1.1 ± 0.0	0.3 ± 0.0	259.2 ± 2.1
	Mid section	1.2 ± 0.1	726.4 ± 5.3	26.3 ± 0.5	70.2 ± 3.5	4.5 ± 0.0	0.3 ± 0.0	267.9 ± 4.3
	Distal	3.1 ± 0.4	813.2 ± 5.5	41.2 ± 0.2	84.9 ± 5.2	2.2 ± 0.1	0.4 ± 0.0	219.8 ± 1.5
FHIA 19	Proximal	1.6 ± 0.0	982.5 ± 4.6	33.1 ± 0.1	61.5 ± 3.4	2.6 ± 0.0	0.3 ± 0.0	218.4 ± 2.2
	Mid section	3.0 ± 0.1	1013.0 ± 5.3	40.4 ± 0.4	72.1 ± 5.6	4.1 ± 0.1	0.3 ± 0.0	237.2 ± 2.4
	Distal	3.1 ± 0.1	1013.8 ± 5.6	42.3 ± 0.2	74.9 ± 4.3	3.6 ± 0.0	0.3 ± 0.0	259.6 ± 3.2
Apantu pa	Proximal	2.1 ± 0.1	772.0 ± 4.4	13.4 ± 0.1	84.2 ± 4.4	1.1 ± 0.0	0.3 ± 0.0	159.1 ± 2.2
	Mid section	2.1 ± 0.1	769.3 ± 6.7	14.3 ± 0.1	85.9 ± 2.6	1.1 ± 0.0	0.3 ± 0.0	162.0 ± 2.4
	Distal	3.0 ± 0.1	773.2 ± 4.4	18.2 ± 0.2	84.2 ± 5.4	1.15 ± .07	0.28 ± .02	157.01 ± 2.2

Mean values (mg/100 g, dry matter basis) from triplicate analysis ± standard deviation

### Starch granules and cell examination

**Fig. 1:**
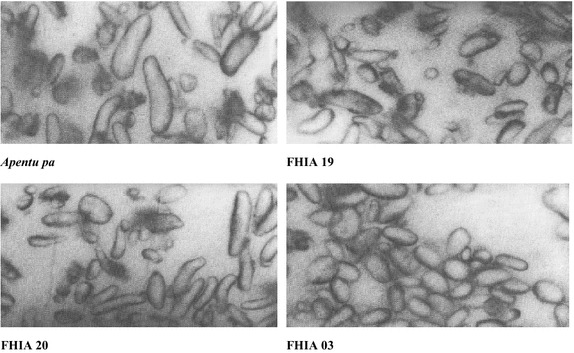
Starch granule morphology of plantain and cooking banana cultivars (Mag. × 145)

**Fig. 2:**
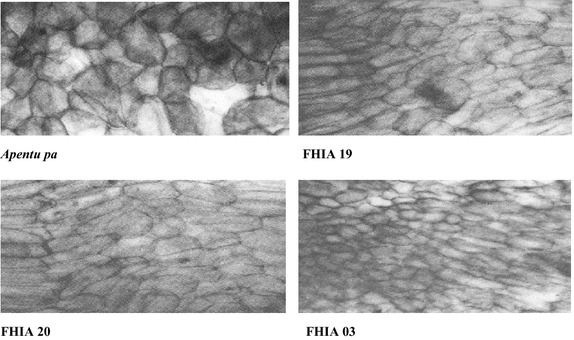
Starch cell morphology of plantain and cooking banana cultivars (Mag. × 145)

The largest starch granule size was found in *Apantu pa* plantain, followed by FHIA 19 and then FHIA 20. The FHIA 03 cooking banana had the smallest granule sizes (Figs. 1, 2). There was also a gradual decrease in starch granule size from the proximal sections to the distal sections of all the cultivars. Whereas the *Apantu pa* consisted predominantly of one type of granules, which were mostly plate like in nature with a few being irregular shaped ones, FHIA 19 and FHIA 20 plantain hybrids consisted of two types of granules: longitudinal and plate-like which were arranged concurrently. FHIA 03 was also composed predominantly of two types of granules: longitudinal and rounded cells with each type of granules grouped together. The starch granule sizes of the cultivars were assessed to find out if there were possible differences among the cultivars. The largest starch granule size was found in Apantu pa plantain. The observed differences in the starch granule sizes of the cultivars may be due to their genetic background. The starch granule size and shape of the hybrids, which were crosses between exotic bananas and plantains varied considerably from that of the landrace. The amylose content has been found to be was relative to the granular morphology (Delpeuch et al. 1978). Small granules have the lowest amylose content whiles the large ones have the highest.

## Conclusions

The new and improved hybrids were heavier and had more hands on a bunch compared to the local *Apentu pa* landrace. The *Apentu pa* had more starch and a firmer pulp compared to the new hybrids. All new plantain hybrids compared very well to *Apentu pa* in terms of the pulp to peel ratio. The FHIA 20 plantain hybrid had the most protein and fibre content amongst the four varieties studied. In terms of the mineral composition, the new varieties significantly had more iron and potassium than *Apentu pa*. Considerable variation existed in the starch microstructure of the various cultivars. The shape of *Apantu pa* starch granules consisted predominantly of one type—plate-like to round. The hybrids consisted of two types of granules. Variations in the starch granule size also existed between and within the cultivars. *Apantu pa* had the largest granule size followed by FHIA 19, FHIA 20 and then FHIA 03. Granule size also decreased from the proximal sections to the distal sections.

## Materials and methods

### Sample selection and preparation

Fruit quality characteristics of two tetraploid plantain hybrids FHIA 19 and FHIA 20, a tetraploid cooking banana hybrid FHIA 03 and a triploid local landrace plantain Apantu pa were obtained from Volta River Estates Ltd (V.R.E.L.) experimental farms, Akuse, Ghana and used for the study. The tetraploid plantain hybrids of the genomic group (AAAB) and cooking banana hybrid of the genomic group (AABB) were developed through years of breeding and selection by the *Fundación Hundureña de Investigación Agricola* (FHIA), Honduras. These hybrids have been introduced in Ghana through the Crops Research Institute (CSIR) for testing. The tetraploid plantain hybrids were derived from crosses between triploid plantain landraces (AAB) and exotic diploid bananas (AA). Characteristics of the hybrid genotypes include high yielding, resistance to black sigatoka disease, draught tolerance and are less prone to lodging than plantains. To compare these new varieties to locally available plantain landraces, *Apantu pa* a false horn triploid (AAB) Ghanaian Landrace plantain was used. This was chosen because it is one of the most preferred plantain types grown and traded in Ghana. The *Apantu pa* landrace plantains attract highest market prices due to their desirable bunch characteristics and multipurpose nature. It is however susceptible to Black sigatoka disease.

Five bunches of unblemished fruits from each cultivar were selected at random and harvested. The maturity of fruits chosen for this study was ‘full three-quarters’, meaning that the individual fingers had less prominent angles i.e. fully mature but green: this criterion for maturity is based on the Jamaican practice (Simmonds 1966). The harvesting of bunches was done three times depending on the availability of fruits that reached the required stage of maturity. This means that each variety had 15 bunches for analysis. The harvested bunches were stored at ambient conditions (28–31 °C, 56–62 % RH) on a wooden platform. Twenty fruits at the proximal, midsection and distal hand position from the five bunches were randomly selected, washed and peeled. The peeled fruits were cut into slices of 0.5 cm thick disc using a vegetable slicer (Qualheim-electro-cut, model 101, Qualheim Inc. USA). The slices were then diced, thoroughly mixed thoroughly with a Hobart cutter (model 84142, The Hobart manufacturing Co Ltd, Don Mills Ont. Canada). The sample was freeze-dried using an Edward’s bench freeze-drier (Edwards Instrument Ltd., Hornchurch, Essex, UK). Prior to analysis the freeze-dried samples were ground with a Hammer Mill (Christy and Morris Ltd., England) equipped with a 250 μm sieve. The flour samples obtained were packaged into polypropylene bags and kept under cold storage (4 °C).

### Methods

#### Physical characteristics

##### Bunch weight

The average weight of the bunch was determined on the whole bunch using a Salter and Scale (±0.1 g).

##### Fruit length

Measurements were done on the outermost curvatures of the fruit. An Inextensible measuring tape was used. The fruit length was measured at the proximal, midsection and distal hand positions of the plantain and cooking banana samples.

##### Fruit width

Measurements were done at the widest midpoint of the fruit. An Inextensible measuring tape was used. The fruit width was measured at the proximal, midsection and distal hand positions of the plantain and cooking banana samples.

##### Number of fingers per bunch

The number of hands of fruits on each bunch was counted to ascertain the number of hand on a bunch.

##### Pulp firmness

Fruit samples were washed and 1 cm of fruit pulp was cut transversely at the mid-point perpendicular to the longitudinal axis. The 1 cm pulp disc was peeled and the peak force (g) required to cut completely through the disc/slice was determined. This was done by using a Warner-Blatzler blade connected to a TA-XT2 texture analyzer, (Stable Micro Systems, Halsmere, and Surrey, England) interfaced with an IBM Computer. The 1 cm pulp disc was placed flat surface down onto the horizontal mounting place. The pulp firmness was measured at the proximal, midsection and distal hand positions of the plantain and cooking banana samples using a pre-test speed of 10.0 mm/s, a test speed of 5.0 mm/s, a post-test speed of 10.0 mm/s, a distance of 20.0 mm, a force threshold of 20.0 g and a contact force of 5.0 g.

##### Pulp colour

Colour was measured with a Minolta Colour Meter Model CR-300 (Minolta Camera Co. Ltd. Inc., Tokyo, Japan) using a white porcelain plate with L = 98.0, a = −0.20, and b = 1.65 as reference. Results were expressed in Hunter L a* and b* values. Three random readings per sample were obtained and averaged. The measuring head was placed on the pulp surface and readings taken in triplicates.

##### Pulp to peel ratio

This was done on weight basis. Pulp and peel weights were determined using a Mettler Toledo AG240 electronic balance (±0.1 mg). The pulp-to-peel ratio was calculated from the pulp and peel weights using the formula:

$${\text{Pulp}}\;{\text{to}}\;{\text{peel}}\;{\text{ratio}} = {\text{PW}}/{\text{FW}} - {\text{PW}}$$

where FW = fruit weight, PW = pulp weight.

##### Proximate composition

The moisture, crude protein (N × 5.7), fibre and ash contents were determined by Association of Official Analytical Chemists Approved methods 925.10, 920.87, 920.86 and 923.03 respectively (AOAC 1990).

##### Starch content

Starch content was determined using the modified ferricyanide (acid hydrolysis) method (Bainbridge et al. 1996).

### Mineral analysis

#### Wet digestion of sample

The first step involved in the elimination of the inorganic materials through the procedure of wet ashing. About 0.5 g of the sample was weighed into a 250 ml beaker. Twenty-five ml (25 ml) of concentrated nitric acid was added and beaker covered with a watch glass. The sample was digested with great care on a hot plate in a fume chamber until the solution was pale yellow. The solution was cooled and 1 ml perchloric acid (70 % HClO_4_) added. The digestion was continued until the solution was colourless or nearly so (the evaluation of dense white fumes indicates the removal of nitric acid). When the digestion was completed, the solution was cooled slightly and 30 ml of distilled water added. The mixture was brought to boil for about 10 min and filtered hot into a 100 ml volumetric flask using a Whatman No. 4 filter paper. The solution was then made to the mark with distilled water.

#### Determination of Ca, Mg, Zn and Fe

One ml of the digest was used to determine the Ca, Mg, Zn and Fe of the sample using the Perking Elmer Precisely A Analyst 400 Atomic Absorption Spectrophotometer with an acetylene flame. The AAS was fitted with Zn and Fe EDL lamps and Mg and Ca CHCL lamps set at wavelengths of 213.86 λ, 248.33 λ, 285.21 λ and 422.67 λ respectively.

#### Determination of Na and K

Two (2) ml of the digest was used in the determination of sodium and Potassium using the flame photometric method. The photometer (Jenway, United Kingdom) model PF P7 with methane gas was used.

#### Phosphorus determination

Two (2) ml aliquot of the digest was reacted with 5.0 ml molybdic acid (The molybdic acid was prepared by dissolving 25 ml of ammonium molybdate in 300 ml distilled water; with 75 ml of concentrated sulphuric acid in 125 ml of water to get 0.5 l of molybdic acid) 1 ml each of 1 % hydroquinone and 20 % sodium sulphite was added in that sequence, and the solution was made up to 100 ml and allowed to stand for 30 min in order to allow the colour to stabilize after which the absorption was measured at 680 nm. A standard curve colorimetric reading versus concentration of phosphorus using portions of standard phosphorus solutions (1, 2 and 3 ml) subjected to reactions with molybdic acid, hydroquinone and sodium sulphate solutions was drawn. All readings were corrected by the reading of a blank to eliminate the effect of any colour produced by the reagents.

### Starch granules and cell examination

#### Identification of starch granules

Dried, ground samples were used for the examination of the starch granules. A minute quality of the sample was added to a small drop of water on a slide and thoroughly mixed taking care not to break any air bubbles. The mixture is then covered with cover slip. Excess water was removed by means of a filter paper and a little dilute iodine was run under the cover slip. Microscopical examination was done using a TMS-F Light Microscope and photomicrographs of the slide taken using a Nikon camera (Nikon Co., Tokyo, Japan) attached to the microscope at a magnification of 145.

### Examination of starch cells

#### Sample preparation

Fruit pulp kept under kept under ambient conditions (28–31 °C) were examined. The samples were washed peeled and pulp of dimension 7.5 mm × 5 mm × 5 mm were sectioned using a dissection blade. The slides were cut along the transverse section and the tissues were then examined for their starch microstructure.

#### Fixation and dehydration

Ten milliliters of formalin (4 % of 40 % commercial formalin) was taken and 0/9 g of pure sodium chloride (Analar grade) added. Distilled water (100 ml) was added and the resulting solution stirred for the dissolution of the sodium chloride. The resulting solution (formol-saline) was adjusted to pH 7.6 with dilute NaOH and/or hydrochloric acid and used as the fixative. The cut tissues were placed in the fixative for 24 h, washed with distilled water and dehydrated through a graded series of aqueous alcohol (50, 70, 90 % and absolute alcohol) each for 30 min. The dehydrated tissues were or de-alcoholized in an antemedia (toluene) for 2 h (Mahoney 1973).

#### Embedding

The cleared samples were impregnated with molten paraffin wax-benzene (50/50) mixture for 1 h. Samples were transferred to molten paraffin wax of melting point 58 °C for about 1 h and finally embedded in molten paraffin wax in a mould. The mould was transferred into cold water to solidify the wax (Peacock 1962).

#### Sectioning

Sections of tissues (8 µ) were cut using the laboratory scale sledge microtome (Erma Inc. Tokyo, Japan). Egg albumen solution was spread on the samples slides and the sections floated on it. The slide was warmed on a hot plate sufficiently to soften but not melt the paraffin and any fold in the section flattened out. The water was drained off and the slide left on the hot plate to dry.

#### Staining and examination

The fixed tissues on the slides were immersed in Xylene to remove the wax. The dewaxed samples were passed through a series of ethanol (absolute ethanol, 90 and 70 %). After this treatment, the samples were stained in safranin for 10 min, washed again in a series of ethanol (50, 70 % and absolute) and counterstained in Fast green for 1 min. The samples were then cleared in clove oil and mounted in Euparol (Flatters and Garvett Ltd., England). Examination of starch cells were done using a TMS-F light Microscope and photomicrographs of the slide taken using a Nikon camera (Nikon Co., Tokyo, Japan) attached to the microscope at a magnification of 145.

### Statistical analysis

Data from the above analysis were subjected to statistical analysis using Statgraphics (Statgraphics Plus 3.0 for Windows, Rockville, USA). Descriptive statistics was done to describe the data. ANOVA was used to determine whether significant differences (p < 0.05) exist between the different varieties.
